# Paternal occupational contact level and childhood leukaemia in rural Scotland: a case-control study

**DOI:** 10.1054/bjoc.2001.1694

**Published:** 2001-04

**Authors:** L J Kinlen, S Bramald

**Affiliations:** Department of Public Health, CRC Cancer Epidemiology Research Group, University of Oxford, Radcliffe Infirmary, Oxford, OX2 6HE, UK

**Keywords:** childhood leukaemia, childhood non-Hodgkins lymphoma, infection, paternal occupational contacts, rural population mixing, Scotland

## Abstract

In a national Scottish study of 809 cases of leukaemia and non-Hodgkins lymphoma diagnosed in 1950–89 among children aged 0–4 years who were born in Scotland, together with 2363 matched population controls, we investigated one aspect of the infective hypothesis. This concerns whether in rural areas (where the prevalence of susceptible individuals is likely to be higher) the risk is greater among the young children of men whose work involves contacts with many different people, particularly children, as noted in certain childhood infections. A positive trend was found in rural areas across 3 levels of increasing paternal occupational contact (as recorded at birth) by each of 2 previously defined classifications; no such effect was found in urban areas. The rural trend was more marked in that part of the study period with greater population mixing, but the difference from the period with less mixing was not itself significant, leaving open whether these rural findings reflect the extreme isolation of much of rural Scotland, or the effects in such areas of a degree of population mixing. In marked contrast, among the 850 cases and 2492 controls aged 5–14, those in rural areas in the higher population mixing period showed a significantly *decreasing* trend with increasing paternal occupational contact level. This would be consistent with immunity produced either by earlier infection at ages 0–4 years, or directly by low doses of the infective agent that were largely immunizing at these older ages. The findings overall provide further support for infection underlying childhood leukaemia and for the role of adults.© 2001 Cancer Research Campaign http://www.bjcancer.com


*British Journal of Cancer* (2001) **84**(7), 1002–1007
© 2001 Cancer Research Campaign
doi: 10.1054/ bjoc.2001.1694, available online at http://www.idealibrary.com on IDEAL®

http://www.bjcancer.com

### Paternal occupational contact level and childhood leukaemia in rural Scotland: a case–control study

**LJ Kinlen and S Bramald**

CRC Cancer Epidemiology Research Group, Department of Public Health, University of Oxford, Radcliffe Infirmary, Oxford OX2 6HE, UK

**Summary** In a national Scottish study of 809 cases of leukaemia and non-Hodgkins lymphoma diagnosed in 1950–89 among children aged 0–4 years who were born in Scotland, together with 2363 matched population controls, we investigated one aspect of the infective hypothesis. This concerns whether in rural areas (where the prevalence of susceptible individuals is likely to be higher) the risk is greater among the young children of men whose work involves contacts with many different people, particularly children, as noted in certain childhood infections. A positive trend was found in rural areas across 3 levels of increasing paternal occupational contact (as recorded at birth) by each of 2 previously defined classifications; no such effect was found in urban areas. The rural trend was more marked in that part of the study period with greater population mixing, but the difference from the period with less mixing was not itself significant, leaving open whether these rural findings reflect the extreme isolation of much of rural Scotland, or the effects in such areas of a degree of population mixing. In marked contrast, among the 850 cases and 2492 controls aged 5–14, those in rural areas in the higher population mixing period showed a significantly *decreasing* trend with increasing paternal occupational contact level. This would be consistent with immunity produced either by earlier infection at ages 0–4 years, or directly by low doses of the infective agent that were largely immunizing at these older ages. The findings overall provide further support for infection underlying childhood leukaemia and for the role of adults. © 2001 Cancer Research Campaign http://www.bjcancer.com

**Keywords**: childhood leukaemia; childhood non-Hodgkins lymphoma; infection; paternal occupational contacts; rural population mixing; Scotland

Significant increases of childhood leukaemia have repeatedly been recorded in situations of marked rural–urban population mixing, confirming the hypothesis that this disease has an underlying infective basis. For the increase in population density implicit in such mixing would increase the level of contacts between susceptible (more prevalent in rural areas) and infected individuals, thereby promoting an epidemic of the relevant infection. The infective agent in question is unknown, but the absence of marked space-time clustering of childhood leukaemia in the general population indicates that it must, as in known infection-based malignancies, be an uncommon response i.e. that the infection itself is largely immunizing. Adults as well as children appear to be involved in its transmission (Kinlen et al, 1993a; Kinlen, 1995).

In the case of young (largely pre-school) children aged 0–4 years, an important contribution to their overall level of direct and indirect contacts will be made by fathers' contacts at work. 'High contact' occupations have already been associated with an increased incidence of childhood cytomegalovirus infection and paralytic poliomyelitis. Furthermore, in situations of extreme rural population mixing which had produced excesses of childhood leukaemia, risk was greater among the young children of those whose work involved many community contacts (Kinlen, 1997). No such increases have been found in mortality studies of the question in the (largely urban) general population of England and Wales (Kinlen, 1997; Fear et al, 1999). As an urban population would be expected to have the raised levels of immunity to infective agents often associated with high population density, we have considered the question still open as to whether high levels of paternal occupational contacts have effects on the *incidence* of childhood leukaemia in young children in a very rural area, where the prevalence of susceptible individuals would be unusually high.

We have therefore investigated paternal occupational contact level in relation to childhood leukaemia in a national case–control study in Scotland in the period 1950–89, with particular reference to rural areas and the possible role of population mixing.

*Received 16 October 2000*
*Revised 15 January 2001*
*Accepted 17 January 2001*

*Correspondence to:* LJ Kinlen

### METHODS

### Cases and controls

Eligible cases consisted of children (aged 0–14) diagnosed in Scotland with leukaemia and non-Hodgkin's lymphoma (NHL) in the years 1950–89. For each child for whom a birth certificate was traced in Scotland that showed a parental occupation, 3 controls of the same age and sex were randomly chosen from the birth registers of the same county. A high proportion of the cases and controls had been included in an earlier investigation of paternal preconceptional occupational exposure to radiation (Kinlen et al, 1993b which gives further details of control selection). Young children (aged 0–4 years) were the main focus of our study, since in the case of older children, a change of paternal occupation is more likely between birth and diagnosis; nevertheless, older children were also investigated. So that the study was truly independent of previous work, we excluded cases that were covered in a previous study of paternal occupational contact levels within excesses of childhood leukaemia associated with marked rural population mixing; these consisted of residents of Glenrothes new town and of the rural home areas of certain North Sea oil industry workers, together with residents of Highland counties with many hydro-electric schemes and of areas near other large rural construction projects (Kinlen, 1997).

### Parental occupations

Details of paternal occupation as recorded on the birth certificate were abstracted for all the children eligible for inclusion in the study, except for a few cases and controls in which the mother's occupation was used, the father not being mentioned. These occupations were coded to the 1960 occupational classification of the Registrar General and then allocated to one of 3 categories of estimated contact level according to 2 definitions that were found to be informative in a previous study of paternal occupational contact levels and childhood leukaemia (Kinlen, 1997). The largest category (the reference) was common to both sets of definitions, but whereas by one set the middle and highest categories each had substantial numbers of cases and controls, by the other definition the highest category had much smaller numbers. We have therefore preferred the former, the categories being defined as follows:

#### *Low, medium- and indeterminate-contact level*

Agricultural occupations alone were classified to a low-contact category, while all occupations other than those in the 2 categories described below were regarded as being in a medium category, including those of uncertain contact level. These 3 categories together formed the reference group in this study.

#### *High-contact level*

This category included: salesmen, students, doctors, nurses and the providers of other services directly to many different people.

#### *Very-high-contact level*

People in occupations involving prolonged and relatively close contact with children, the age group with the greatest experience of new infections, are likely to have an unusually high level of contacts with the infection postulated as underlying leukaemia. Teaching and other occupations carried out in educational establishments (taken as those with the words 'child', 'school', 'college' or 'nursery' in the title) were therefore allocated to a very-high-exposure category, as in a study of cytomegalovirus infection (Stagno et al, 1984). Also included here were occupations taking people outside their local community (transport etc.), and those involving regular changes of colleagues and place of work, as in the construction industry (in Scotland, often distant from home). The categories are defined in more detail in the Appendix.

The data were also analysed according to the other set of definitions in which the small group of 'educational' occupations alone formed the very-high-contact category (Kinlen, 1997).

### Rural and urban areas

Urban areas were defined as the postcode districts that embraced the 4 Scottish cities and the central industrial belt, a definition used previously (Kinlen et al, 1993a). Rural areas, which were of primary interest, were the remainder of Scotland.

### Higher and lower population mixing periods

A division of the study into later and earlier periods of, respectively, higher and lower population mixing was facilitated by the growth of the North Sea oil industry in the later period, and by our removal from the earlier period of 5 whole counties with large hydro-electric schemes because of their inclusion in a previous study (see above). Relevant here is the unprecedented scale of the North Sea oil industry, the rural effects of which extended far beyond the areas previously studied (Kinlen et al, 1993a). For this has involved not only the building of oil terminals, platform construction yards and the platforms themselves at remote sites often with large work camps and extensive haulage work, but also the operation of oil rigs and associated support industries, such as supplying the rigs and transportation of workers. All this would have increased the level of rural population mixing in a wide area in the later period (1970–89) of the study as compared with the earlier (1950–69).

The difference between the sub-periods was also deliberately accentuated. The creation of Glenrothes new town in Fife, with rapid population growth from around 1000 in 1948 to over 19 000 in 1966, absorbed much of the available construction workforce of the county, besides indirectly affecting a wide area around Glenrothes from which a large proportion of the residents of the new town had come, and in which they often continued to work (e.g. at the Rosyth naval dockyard) (Kinlen et al, 1990 and unpublished). To increase further the contrast between the two periods, the (rural) county of Fife in the period 1950–69 was included with the higher population mixing period (though Glenrothes itself was already excluded as explained above); the rest of the study formed the lower mixing period.

### Analysis

Matched data on cases and controls were examined separately in rural and urban areas for the 3 categories of increasing paternal contact level using conditional logistic regression with EGRET software (1991), which produces estimates of odds ratios approximating to relative risks, together with confidence intervals. The analyses were repeated after adjusting for social class, categorized as classes I&II, IIIN, IIIM (with armed forces), and IV&V. Evidence of linear trends was examined using a likelihood ratio test (Breslow and Day, 1980).

### RESULTS

Details of paternal occupational contact category were available for a total of 809 cases aged 0–4 and for most (92%) of these, corresponding details were available for each of 3 controls. For 65 cases, only 2 controls were available for analysis because the birth certificate of the third control showed either no parental occupation or an inappropriate county of residence. For this age group, the risk of leukaemia and NHL, adjusted for social class, showed a significant positive trend with increasing paternal contact level in rural ($P$ = 0.02) but not in urban ($P$ = 0.26) areas (Table 1); the difference between these trends is statistically significant ($P$ = 0.013). The corresponding analysis using the other set of definitions, with teachers alone in the very-high-contact category (see Methods) also showed a significant trend in rural ($P$ = 0.04) but not in urban ($P$ = 0.61) areas (data not shown). The effects of paternal occupation in rural areas appeared more marked ($P$ for

© 2001 Cancer Research Campaign

**Table 1** Childhood leukaemia and NHL at ages 0–4 by paternal occupational contact category and urban–rural status at birth: numbers and odd ratios

| Rural<br>Contact category | Cases | Controls | OR[a] | 95% CI |
|---|---|---|---|---|
| Medium and low | 172 | 563 | 1.00 | |
| High | 50 | 143 | 1.03 | (1.69, 1.52) |
| Very high | 94 | 218 | 1.43 | (1.04, 1.95) |
| | Trend (*P* value) | 0.02 | | |
| **Urban<br>Contact category** | **Cases** | **Controls** | **OR[a]** | **95% CI** |
| Medium and low | 281 | 799 | 1.00 | |
| High | 86 | 220 | 1.05 | (0.77, 1.42) |
| Very high | 126 | 420 | 0.88 | (0.68, 1.13) |
| | Trend (*P* value) | 0.26 | | |

[a]Adjusted for social class.

**Table 2** Childhood leukaemia and NHL at ages 0–4 by paternal occupational contact category and urban–rural status at birth in higher and lower population mixing (PM) periods: numbers and odds ratios

| Rural | Higher PM period | | | | Lower PM period | | | |
|---|---|---|---|---|---|---|---|---|
| Contact category | Cases | Controls | OR[a] | CI | Cases | Controls | OR[a] | CI |
| Medium and low | 92 | 317 | 1.00 | | 80 | 246 | 1.00 | |
| High | 31 | 94 | 0.86 | (0.51, 1.46) | 19 | 48 | 1.25 | (0.68, 2.30) |
| Very high | 59 | 127 | 1.58 | (1.05, 2.39) | 35 | 91 | 1.20 | (0.74, 1.96) |
| | Trend (*P* value) | | 0.02 | | Trend (*P* value) | | 0.45 | |
| **Urban** | **Higher PM period** | | | | **Lower PM period** | | | |
| **Contact category** | **Cases** | **Controls** | **OR[a]** | **CI** | **Cases** | **Controls** | **OR[a]** | **CI** |
| Medium and low | 119 | 357 | 1.00 | | 162 | 442 | 1.00 | |
| High | 47 | 120 | 1.11 | (0.72, 1.72) | 38 | 100 | 0.95 | (0.61, 1.49) |
| Very high | 60 | 194 | 0.96 | (0.66, 1.40) | 66 | 226 | 0.82 | (0.58, 1.15) |
| | Trend (*P* value) | | 0.75 | | Trend (*P* value) | | 0.20 | |

[a]All odds ratios adjusted for social class.

trend = 0.02) in the higher mixing period than in the lower (*P* = 0.45) (Table 2), though the difference between these trends is not statistically significant. The corresponding *P* values for the trends using the other definitions were 0.09 and 0.24, respectively, again not significantly different from each other. No significant trend was found in urban areas in either period (Table 2), or using the other definitions.

Although younger children were the main focus of interest, 867 cases and 2546 matched controls at ages 5–14 were also investigated. In contrast to the positive relation found among younger children, these older children showed (see Table 3A) a significantly declining trend in relative risk with increasing contact category in rural areas in the higher population mixing period (*P* for trend = 0.04). Using the other category definitions, the trend was again downward and significant (*P* = 0.02), with no case and 14 controls in the very-high (teachers only) category (Table 3B). The difference between these trends at ages 0–4 and 5–14 years was, by each definition, statistically significant ($P < 0.002$ and $< 0.005$). No significant trend (in either direction) was found in rural areas in the lower population mixing period, nor in urban areas in either periods, whichever definition of contact category was used.

### DISCUSSION

Occupational risks of infection are well known and there is nothing novel in evidence of transmission of infection from adult to child, including in childhood leukaemia (Kinlen, 1995). The notion of an increased risk of infection among the children of people in certain occupations is less familiar. In cytomegalovirus infection, however, an increased risk is well established among the children of both nursery workers and fathers with occupations, or leisure activities, involving much contact with children (Stagno et al, 1982, 1984; Adler, 1988; Yow et al, 1988; Murph et al, 1991). Certain epidemics of paralytic poliomyelitis also occasioned similar observations (Cowan, 1950; Logan, 1953; McFarlan et al, 1946), while a study of poliovirus antibody levels in relation to social factors found that a higher than average level for an individual's social class was often associated with a high-contact occupation (Backett, 1957). Our findings in rural areas of Scotland imply that *in certain circumstances* leukaemia at very young ages can likewise be affected by levels of paternal occupational contacts. They cannot, as suggested by Fear et al (1999), be attributed to social class as this was adjusted for in the analysis, though

 *© 2001 Cancer Research Campaign*

**Table 3** Childhood leukaemia and NHL at ages 5–14 in rural areas at birth by paternal occupational contact category (by 2 definitions) in higher population mixing period: numbers of cases and controls and odd ratios

| A Contact category | Cases | Controls | OR[a] | 95% CI |
|---|---|---|---|---|
| Medium | 128 | 326 | 1.00 | |
| High | 34 | 113 | 0.81 | (0.51, 1.29) |
| Very high | 47 | 178 | 0.73 | (0.49, 1.08) |
| | | Trend (*P* value) | 0.04 | |

| B Contact category[b] | Cases | Controls | OR[a] | 95% CI |
|---|---|---|---|---|
| Medium | 128 | 326 | 1.00 | |
| High | 81 | 277 | 0.79 | (0.56, 1.10) |
| Very high | 0 | 14 | 0.00 | (0.0, 0.70) |
| | | Trend (*P* value) | 0.02 | |

[a]Adjusted for social class.
[b]Other definitions, with educational occupations only in the very high category.

its effects on risk were slight. The findings therefore add to the evidence from many population mixing studies (reviewed in Kinlen, 1995; Doll, 1999), including a recent cohort study (Kinlen and Balkwill, 2001), that strongly support an infective basis in childhood leukaemia.

Young children (aged 0–4 years) were of primary interest in the present study. For among such children, not only will paternal occupation at birth have had less opportunity to change by time of diagnosis than in the case of older children, but paternal contacts make a larger contribution to a younger child's total contacts, before school becomes the dominant influence. It is not implied, of course, that occupations as recorded on birth certificates are only relevant at the time of birth; the timing of any infective transmission will be determined by the timing of the relevant contact at work and, no doubt, the play of chance. We have no means of knowing, however, whether this involves a single or repeated contact, though the latter might be more likely if a large dose of the relevant agent is important (as it is in feline leukaemia).

It needs to be stressed that the present study did not focus on areas of marked population mixing, still less areas that turned out to show significant excesses of childhood leukaemia, as have many of our studies. Instead, a whole country was covered and specifically the extensive rural part of Scotland, amounting to over 73 000 $km^2$, 95% of its land mass. The paternal occupational effects were not associated with any appreciable increase in overall incidence in the rural areas of Scotland, the high-contact categories being too small to do this. That the risk of childhood leukaemia should vary in subgroups within situations falling well short of intense population mixing is not remarkable; it accords with the conventional view that open populations are made up of innumerable, but often interlocking, subgroups differing in proportions of immune and susceptible individuals, as well as in intimacy of contact, and that these differences are important in the spread of infection (Fox et al, 1971).

The paternal occupational effects seen in rural Scotland (see Table 1) were not evident in childhood leukaemia mortality data for rural areas of England and Wales (Kinlen, 1997) raising the question of whether this difference reflects the greater isolation of much of rural Scotland, or the effects there of more recent population mixing. The paternal effects did indeed appear to be stronger (*P* for trend = 0.02) in the higher mixing period (dominated by North Sea oil industry) than in the lower period (*P* = 0.45), as shown in Table 2, though the difference between these trends is not statistically significant. The question about the role of population mixing in relation to these occupational effects therefore remains open, but investigation in countries with appreciable rural populations, as in Scandinavia, should shed light on this question.

It may be noted that all the major occupational groups in the very-high-contact category have independently been linked with infections (including some that are oncogenic) either in the individuals themselves or in their spouses. Besides the anecdotes that abound of upper respiratory infections in teachers on starting or returning to work, their occupational risk of hepatitis B infection is now recognized under the Industrial Injuries Act. Furthermore, the wives of men in jobs requiring frequent absences from home, such as long-distance lorry drivers and construction workers, have long been associated with a high incidence of cervical cancer, a neoplasm caused by human papilloma virus infection. It does not seem unreasonable, therefore, to suppose that such men may also be exposed outside their communities to non-sexually transmitted infective agents or strains.

The restriction of the occupational relationship to rural areas is consistent with the findings of most studies of population mixing and childhood leukaemia (reviewed in Kinlen, 1995). The lower population density in such areas results in a higher prevalence of susceptible individuals, making them more vulnerable to infective outbreaks triggered by new contacts with infected people. Thus, measles mortality in adults has been much higher in rural than in urban Scotland even over the past 50 years. The higher prevalence of immune individuals in urban areas makes them relatively resistant to such outbreaks and here, the pattern of infection underlying childhood leukaemia is more likely to be sporadic. In keeping with this, no effect of paternal occupation contact levels was found in leukaemia mortality data for urban areas of England and Wales (Kinlen, 1997) or for the (largely urban) country as a whole (Fear et al, 1999).

Of note is the significantly *declining* risk of leukaemia at ages 5–14 with increasing level of paternal occupational contacts (see

*© 2001 Cancer Research Campaign* 

Table 3) in rural areas in the higher mixing period – including an intriguing absence of cases in teachers' children born in those areas at these times. These declining trends seem unlikely to be due to chance. Examination of urban and rural areas, higher and lower mixing periods by the two contact category definitions produce 8 subgroups; of these, only 2 showed declining trends at ages 5–14 and these same 2 sub-groups showed a significantly increasing trend at ages 0–4. None of the other 6 subgroups showed a significant trend (in either direction). The declining trend at ages 5–14 years may reflect immunity produced either by earlier infection at ages 0–4 years, or directly by low doses that are immunizing at these older ages, as suggested for similar observations in childhood leukaemia in new towns (Kinlen et al, 1990). The declining risk across the occupational categories at 5–14 years contrasts with the increased incidence at these ages observed in studies that have focused on particular areas affected by marked urban–rural population mixing (Kinlen, 1995), but neither of these is a feature of the present study. It seems probable that these findings reflect differences in the extent of exposure (i.e. in degree of mixing), perhaps mediated by differences in dose of the relevant agent (or agents).

Greaves (1988) has proposed that the common type of acute lymphoblastic leukaemia in the childhood 'peak' ages of 2–6 years, when lymphocytes are actively dividing, is caused by non-specific infections; such infections must increasingly have been deferred from infancy during this century as a result of improved hygiene, and could, he considers, trigger spontaneous mutations in lymphocytes. It is not clear, however, that this hypothesis would readily explain a *deficiency* of leukaemia at older ages.

### Contact categories

Any study of contact levels based only on occupational titles, as in this study, is necessarily crude. On the other hand, many titles were included in the large reference (low and medium contact) category, not because our advisors considered them as of low or medium level (see Kinlen, 1997), but in order to conservatively lean against the hypothesis. Thus occupations of uncertain (and therefore possibly high) contact levels were included here, as well as others such as secretary which would embrace more than one contact level. It is not claimed that our categories would necessarily be appropriate in other situations and another study might have additional details that would allow a different and more sensitive definition of, say, the very-high category. Also, in rural Scotland, with a chronic lack of local employment, the construction industry may be relatively unusual in often involving work away from home on large projects.

We recognize the limited scope of this study of paternal contacts in relation to childhood leukaemia. Paternal occupation represents only one of the opportunities for fathers (and indirectly for their children) to have contacts with a wider infective pool; our study also takes no account of maternal occupational contacts, or the social and recreational contacts of either parent. A study of all parental contacts in rural areas might therefore have found a stronger effect than we detected here. Case–control studies of children in rural areas covering the totality of their direct and indirect contacts would be the ideal, though appropriate measures of overall contacts would be needed for epidemiological purposes.

### REFERENCES

Adler SP (1988) Molecular epidemiology of cytomegalovirus: Viral transmission among children attending a day care center, their parents, and caretakers. *J Ped* **112**: 366–372

Backett EM (1957) Social patterns of antibody to poliovirus. *Lancet* **1**: 778–783

Breslow NE and Day NE (1980) *Statistical Methods in Cancer Research. Vol. 1. The Analysis of Case-Control Studies*. Lyons: International Agency for Research on Cancer (IARC Scientific Publications no. 32)

Cowan HK (1950) *Report of the Medical Officer of Health for Essex for the Year 1949*, 66–73.

Doll R (1999) The Seascale Cluster: a probable explanation. *Br J Cancer* **81**: 3–5

Fear N, Roman E, Reeves G and Pannet B (1999) Are the children of fathers whose jobs involve contact with many people at an increased risk of leukaemia? *Occup Environ Med* **56**: 438–442

Fox JP, Elveback L, Scott W, Gatewood L and Ackerman E (1971) Herd immunity: basic concept and relevance to public health immunization practices. *Amer J Epidemiol*. **94**: 179–189

General Register Office (1960) *Classification of Occupations, 1960* London: HMSO

Greaves M (1988) Speculations on the cause of childhood acute lymphoblastic leukaemia. *Leukaemia* **2**: 120–125

Kinlen LJ (1995) Epidemiological evidence of an infective basis for childhood leukaemia. *Br J Cancer* **71**: 1–5

Kinlen LJ (1997) High-contact paternal occupations, infection and childhood leukaemia: five studies of unusual population-mixing of adults. *Br J Cancer* **76**: 1539–1545

Kinlen LJ and Balkwill A (2001) Infective aetiology in childhood leukaemia: a cohort study of wartime population mixing in wartime Orkney and Shetland. *Lancet* **357**: 858

Kinlen LJ, Clarke K and Hudson C (1990) Evidence from population mixing in British New Towns 1946–85 of an infective basis for childhood leukaemia. *Lancet* **336**: 577–582

Kinlen LJ, O'Brien F, Clarke K, Balkwill A and Matthews F (1993a) Rural population mixing and childhood leukaemia: effects of the North Sea oil industry in Scotland, including the area near Dounreay nuclear site. *BMJ* **306**: 743–748

Kinlen LJ, Clarke K and Balkwill A (1993b) Paternal preconceptional radiation exposure in the nuclear industry and leukaemia and non-Hodgkin's lymphoma in young people in Scotland. *BMJ* **306**: 1153–1158

Kinlen LJ, Dickson M and Stiller CA (1995) Childhood leukaemia and non-Hodgkin's lymphoma near large rural construction sites, with a comparison with Sellafield nuclear site. *BMJ* **310**: 763–768

Logan JS (1953) Poliomyelitis in Southend-on-Sea in 1952. *Proc Royal Soc Med* **46**: 37–41

McFarlan AM, Dick GWA and Seddon HJ (1946) The epidemiology of the 1945 outbreak of poliomyelitis in Mauritius. *The Quarterly J Med* (New Series) **XV**: 183–208

Murph JR, Baron JC, Kice Brown C, Ebelhack CL and Bale Jr JF (1991) The occupational risk of cytomegalovirus infection among day-care providers. *JAMA* **265**: 603–608

Stagno S, Pass RF, Dworsky ME, Henderson RE, Moore EG, Walton PD and Alford CE (1982) Congenital Cytomegalovirus Infection. The relative importance of primary and recurrent maternal infection. *New Engl J Med* **306**: 945–949

Stagno S, Cloud G, Pass RF, Britt WJ and Alford CA (1984) Factors associated with primary cytomegalovirus infection during pregnancy. *J Med Virol* **13**: 347–353

Yow MD, Williamson DW, Leeds LJ, Thompson P, Woodward RM, Walmus BF, Lester JW, Six HR and Griffiths PD (1988) Epidemiologic characteristics of cytomegalovirus infection in mothers and their infants. *Am J Obstet Gynecol* **158**: 1189–1195

*© 2001 Cancer Research Campaign*

### APPENDIX

**Occupational contact categories by 1960 codes**[a]

| | |
|---|---|
| Low, medium and uncertain | All codes other than those specified below |
| High | Sales etc. 121, 135, 230–235, 239,<br>Service and professional 051, 091, 110, 236, 250–6, 263, 265, 267, 269, 275–6, 280–5, 294, 298–9, 310, 320, 321 |
| Very high | Construction industry 053, 056, 061, 070, 080, 150, 152–154, 171, 172, 187, 273, 288<br>Transport etc 190–8, 203–6, 208, 209, 266.<br>Teachers etc 286, 287 |

**Occupational contact categories by 1980 codes**[a]

| | |
|---|---|
| Low, medium and uncertain | All codes other than those specified below |
| High | 001, 009, 013, 017, 024, 026, 034–5, 037, 039–53, 059–63, 100–6, 108–11, 125, 127–8, 130–9, 144–6, 152–3, 159, 161, 163–5, 174, 186, 210, 256, 299 |
| Very high | 018, 031–3, 038, 085, 087, 093, 095, 123, 150–1, 155, 160, 214, 260, 302–5, 308–9, 311, 313, 316, 318–20, 323–31, 334–5, 337, 338 |

[a]Inadequately described occupations (code 330 in 1960) excluded.

*Note.* In the other set of definitions, only children-linked occupations formed the very high contact category viz: 286, 287 (1960 codes); 031-033, 038, 150, 151 (1980 codes). See text.

*© 2001 Cancer Research Campaign*
